# Two novel Warburg micro syndrome 1 cases caused by pathogenic variants in *RAB3GAP1*

**DOI:** 10.1038/s41439-021-00171-9

**Published:** 2021-10-26

**Authors:** Omid Alavi, Hossein Jafari Khamirani, Sina Zoghi, Afrooz Feili, Seyed Alireza Dastgheib, Seyed Mohammad Bagher Tabei, Jamal Manoochehri, Seyed Mehdi Panahandeh, Majid Kamali, Mehdi Dianatpour

**Affiliations:** 1 Shiraz Institute for Stem Cell and Regenerative Medicine, Shiraz University of Medical Sciences, Shiraz, Iran; 2 Department of Tissue Engineering and Cell Therapy, School of Advanced Technologies in Medicine, Shiraz University of Medical Science, Shiraz, Iran; 3 Department of Medical Genetics, Shiraz University of Medical Sciences, Shiraz, Iran; 4 Comprehensive Medical Genetic Center, Shiraz University of Medical Sciences, Shiraz, Iran; 5 Student Research Committee, Shiraz University of Medical Sciences, Shiraz, Iran; 6 Maternal-fetal Medicine Research Center, Shiraz University of Medical Sciences, Shiraz, Iran; 7 Department of Genetics, Fars Science and Research Branch, Islamic Azad University, Marvdasht, Iran; 8 Stem Cells Technology Research Center, Shiraz University of Medical Sciences, Shiraz, Iran

**Keywords:** Development, Neurological disorders, Diseases of the nervous system

## Abstract

In this study, we detected a novel pathogenic variant and a previously reported variant in *RAB3GAP1* by whole-exome sequencing (NM_001172435.2: c.1552C>T, p.Gln518*; c.1471C>T, p.Arg491*). The first patient is a 3-year-old girl who presented with bilateral congenital cataracts, developmental delay, abnormal craniofacial features, drug-resistant constipation, and corpus callosum hypoplasia. The proband of the second family is a 13-year-old boy who suffers from developmental delay, quadriplegia, intellectual disability, abnormal craniofacial features, and corpus callosum hypoplasia.

Warburg micro syndrome (WARBM) and Martsolf syndrome are rare genetic disorders caused by a deficiency in RAB18 protein (OMIM: #212720; #600118). Initially, they were regarded as separate entities; however, later, it was discovered that they are both caused by RAB18 deficiency^1^. Martsolf syndrome is the milder form of RAB18 deficiency, while Warburg micro syndrome is the more severe form.

Warburg micro syndrome is typically characterized by craniofacial, ophthalmic, genitourinary, skeletal anomalies, and neurologic disorders. Clinical findings in both syndromes are generally the same, with the former being milder than the latter^1–5^.

Here, we present two unrelated patients with WARBM1 caused by nonsense pathogenic variants (NM_001172435.2: c.1552C>, p.Gln518*; c.1471C>T, p.Arg491*) in *RAB3GAP1*, the first patients from Iran (Fig. 1A–D).

**Fig. 1:**
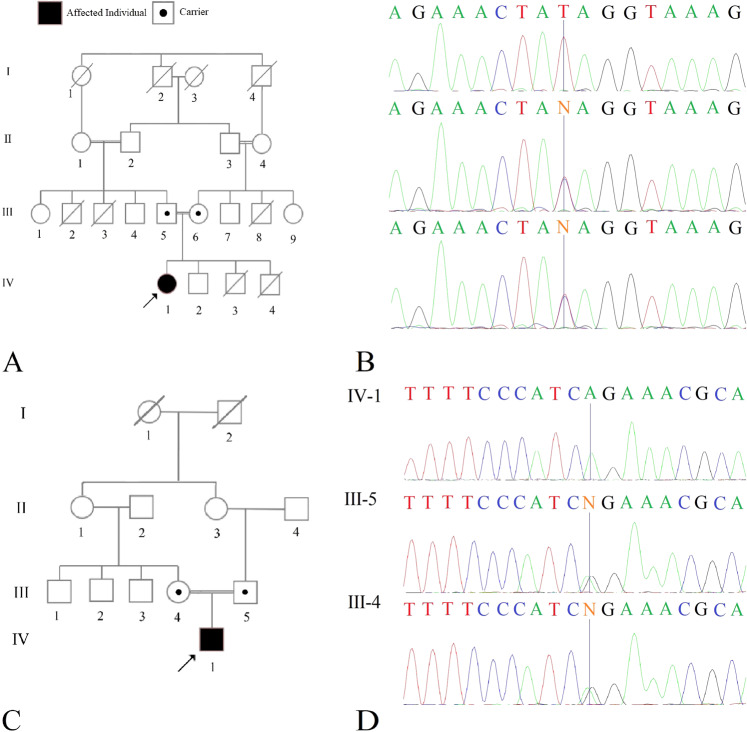
The pedigree and electropherogram of the probands and their parents. **A** The pedigree. **B** The electropherogram of the proband (IV-1), his father (III-5), and his mother (III-6). **C** The pedigree. **D** The electropherogram of the proband (IV-1), his father (III-5), and his mother (III-4).

We present the case of a 3-year-old Iranian girl, the fourth child of consanguineous parents. Their first child is a healthy 9-year-old boy. However, the second and third sons of the family did not survive infancy. The parents reported congenital cataracts and developmental delays in the deceased sons. They died at 5 and 8 months of age (however, it is not clear whether they died due to the same condition). The proband was born following a successful pregnancy at 37 weeks of gestation by caesarian section. At birth, her weight and head circumference were 2700 g and 32.5 cm, respectively. She had bilateral congenital cataracts, which were operated on at Day 35 after delivery. She had developmental delays in motor function, language and speech, and cognitive and social skills. The patient is not able to talk and also sit without support, but her hearing is intact. The patient has not gained weight appropriately. Currently, she weighs 8 kg. In physical examination, lower limb hyperreflexia was noted. She has not started speaking yet. In terms of abnormal craniofacial features, deep-set eyes, a prominent nasal root, a narrow mouth, and large anteverted ears can be recognized. The patient recently suffered from severe constipation and did not respond to medications (Fig. 2).

**Fig. 2:**
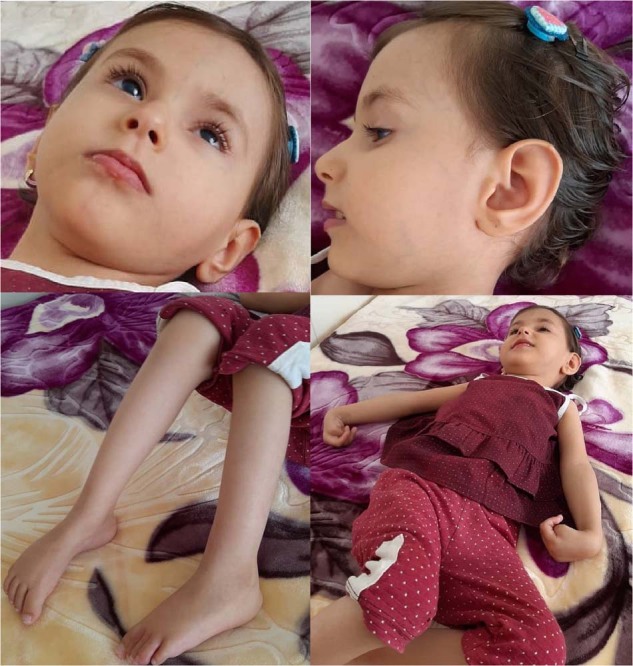
The gross anatomical features of the proband of family 1. Deep-set eyes, a prominent nasal root, a narrow mouth, elbow contracture, and large anteverted ears are present.

Electroencephalography was not remarkable. Eye sonography revealed thick corneas, thin and cataractous lenses, mild irregularity of the inner surface of the posterior aspect of the globe wall, and mild elevation of the optic disc. Brain MRI shows abnormal signal alteration in the periventricular deep white matter with corpus callosum hypoplasia (Supplementary Fig. 1).

The results of routine biochemical tests, including liver, kidney, and thyroid function tests, were normal. TORCH screening test result at birth, complete blood count, and serum electrolytes were normal. The chromosomal analysis revealed a normal karyotype.

Whole-exome sequencing detected a novel nonsense pathogenic variant in *RAB3GAP1* (NM_001172435.2: c.1552C>T, p.Gln518*), which was confirmed by Sanger sequencing.

The same variant was detected in her parents by Sanger sequencing.

The second proband is a 13-year-old Iranian boy, the only child of consanguineous parents (second cousins). He was born at term by normal vaginal delivery. Intrauterine growth restriction was recorded during gestation. At birth, he was 1900 g and 41 cm tall, and his head circumference was 31 cm. He was diagnosed with congenital cataracts. In infancy, he was hypotonic with poor head control. He currently has severe spastic quadriplegia. His language acquisition is minimal. He is in the first Tanner stage of pubertal development. He cannot communicate effectively with his parents and caregivers. Sonographic findings were similar to those of the proband of Family 1. Abnormal craniofacial features in the proband include deep-set eyes, a prominent nasal bridge and root, a maxillary protrusion, and large anteverted ears. Failure to thrive was present during his development course, which did not respond to interventions (Figs. 1C, 3).

**Fig. 3:**
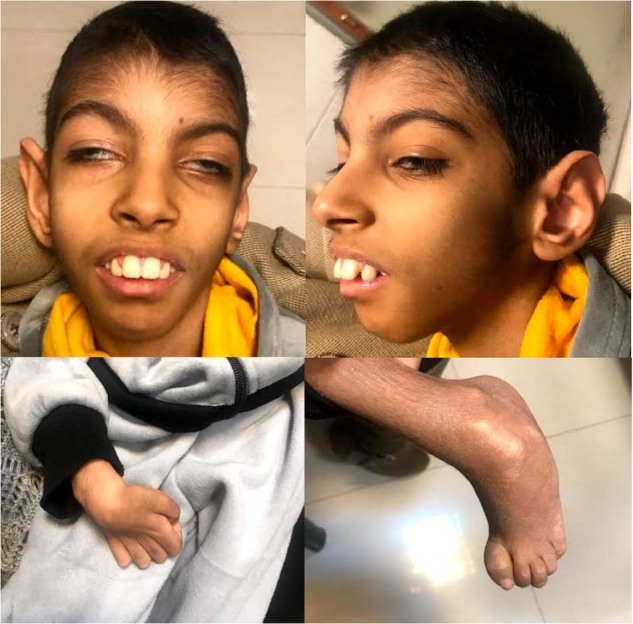
The gross anatomical features of the proband of family 2. Deep-set eyes, a prominent nasal root, a narrow mouth, a maxillary protrusion, elbow contracture, and large anteverted ears are present.

Congenital diaphragmatic hernia (CDH) was detected prenatally and confirmed after delivery. His CDH was repaired on the second day of life. He quickly recovered from the surgery. Lens replacement surgery was conducted when he was six months old. Posterior vitreous detachment was noticed following the surgery. Following the surgery, his sight deteriorated to no light perception.

He had a micropenis, and his testes were not palpable at birth. Increased dehydroepiandrosterone sulfate and decreased luteinizing hormone, follicle-stimulating hormone, and testosterone were recorded in the following months. Sonography was unsuccessful in localizing the undescended testes. Laparoscopic orchiopexy was carried out at 18 months of age; however, the operation was not successful because the testes were not present in the abdominal cavity. Due to abnormal kidney function, he received kidney transplantation at four years of age.

Brain magnetic resonance imaging showed CSF prominence suggesting cerebral atrophy and hypoplasia of the body and splenium of the corpus callosum. Electroencephalography following a few seizure-like episodes at 6 years of age (mild clonus at the right shoulder) failed to show any epileptic activity except in the low-amplitude background (Supplementary Figs. 1D, 2, 3).

TORCH infections were ruled out. Neonatal screening and testing for metabolic disorders were carried out during the first six months of life. No metabolic disorder was detected. He has normal thyroid function. Whole-exome sequencing was conducted for the proband. We found that the proband was homozygous for a variant in *RAB3GAP1* (NM_001172435.2: c.1471C>T, p.Arg491*; rs752667359). The presence of the variant was confirmed by Sanger sequencing. Sanger sequencing detected the same variant in his parents.

None of the variants presented in this study are reported in the gnomAD, Iranome, GME, or TogoVar databases. While p.Arg491* was registered in the ClinVar database, p.Gln518* is novel. p.Gln518* is classified as pathogenic by BayesDel addAF, BayesDel noAF, DANN, EIGEN, EIGEN PC, FATHMM-MKL, FATHMM-XF, LRT, and MutationTaster. Using BayesDel addAF, BayesDel noAF, DANN, EIGEN, EIGEN PC, FATHMM-MKL, LRT and MutationTaster, Arg491* was categorized as pathogenic. Both variants are categorized as pathogenic based on the PVS1, PM2, PP3, and PP5 ACMG classification criteria.

Warburg micro syndrome is a rare autosomal-recessive disorder primarily affecting the visual, nervous, and endocrine systems. Warburg micro syndrome was first described in a Pakistani family presenting with microcornea, microcephaly, congenital cataracts, severe mental retardation, retinal dystrophy, optic nerve atrophy, hypothalamic hypogenitalism, and corpus callosum agenesis^6^. One of the variants detected in the current study was previously described by Morris-Rosendahl et al. in a Turkish patient^7^. Comparing the phenotype of proband 2 of the current study with the previously reported patient, the regressive nature of the development in the patient presented becomes more evident.

RAB18 deficiency was assumed to be the underlying mechanism of Martsolf and Warburg micro syndromes. RAB proteins are a large family of GTPase enzymes that facilitate cell tracking. RAB GTPases typically connect to membranes by binding two carboxy-terminal cysteine residues using their hydrophobic geranylgeranyl groups. Prenylation facilitates their role in synchronizing membrane trafficking^8^. RAB proteins have been shown to have a diverse set of roles in human disorders such as immunodeficiency, cancer, and neurodegenerative disorders^8,9^. Rab18 deficiency can arise from mutations in any of the following genes*: RAB3GAP1, RAB3GAP2, RAB18, or TBC1D20*. Seventy-five percent of RAB18 deficiency cases were attributed to mutations in *RAB3GAP1*^1^.

In this paper, we present the first Iranian patients with pathogenic variants in *RAB3GAP1* causing Warburg micro syndrome. In 2020, Hossein Hozhabri et al. reported two Iranian siblings with Martsolf syndrome caused by a pathogenic variant in *TBC1D20*^10^^.^ The cases described in the study by Hossein Hozhabri et al. and those in the present study are the only RAB18 deficiency cases reported from Iran. These reports suggest that RAB18 deficiency may be underdiagnosed in Iran, requiring a more rigorous approach toward the molecular diagnosis of children with signs and symptoms typical of genetic diseases, including Martsolf and Warburg micro syndrome. Compared with previous reports, one of our patients suffers from severe drug-resistant constipation, which was not mentioned in the literature.

To date, among the 69 different mutations of various types identified in *RAB3GAP1* in 65 families, no mutation hotspot has been recognized. This implies that all 24 exons in each patient have to be studied^1,11,12^. Molecular diagnosis would considerably benefit patients and caregivers. Molecular diagnosis would permit genetic counseling for future children of the family. Furthermore, the clinical course of the disease in the proband, despite the phenotypic variabilities, becomes predictable. Thus, clinical interventions for signs and symptoms such as cryptorchidism and developmental delay can be beneficial.

## HGV Database

The relevant data from this DataReport are hosted at the Human Genome Variation Database at 10.6084/m9.figshare.hgv.3097, 10.6084/m9.figshare.hgv.310.

## Supplementary information

Supplementary Figure 1

Supplementary Figure 2

Supplementary Figure 3

Patient consent form 1

Patient consent form 2

## Data Availability

All data generated or analyzed during this study are included in the final published article.
