# Supplementary material for: Two novel Warburg micro syndrome 1 cases caused by pathogenic variants in RAB3GAP1
Source: Hum Genome Var. 2021 Oct 26;8:39. doi: 10.1038/s41439-021-00171-9 (PMC8548584; doi:10.1038/s41439-021-00171-9)
Supplement: Supplementary file 1 — Supplementary Figure 1 [file 41439_2021_171_MOESM1_ESM.docx]

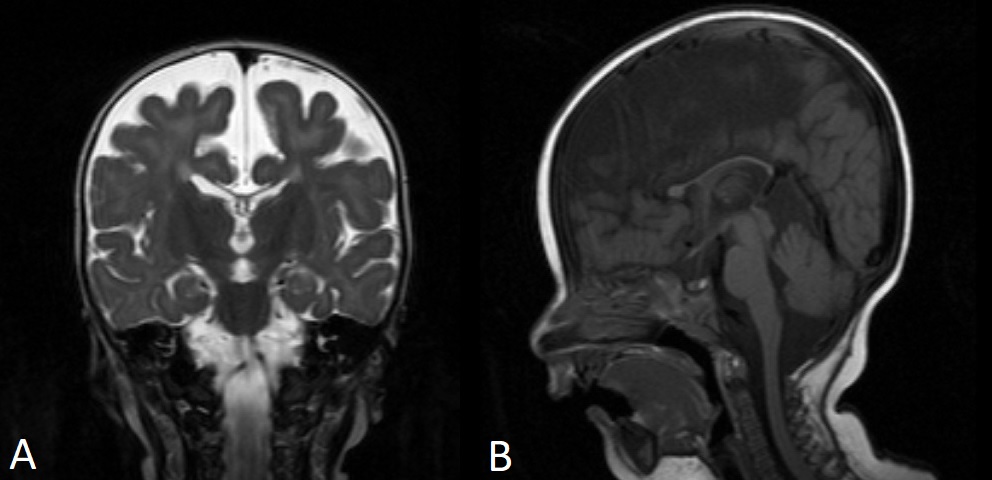


Supplementary Figure 1. Magnetic resonance imaging of the proband of family 1. A; Prominence of cerebrospinal fluid and brain atrophy are noted. B; The corpus callosum is hypoplastic.
