# Supplementary material for: Two novel Warburg micro syndrome 1 cases caused by pathogenic variants in RAB3GAP1
Source: Hum Genome Var. 2021 Oct 26;8:39. doi: 10.1038/s41439-021-00171-9 (PMC8548584; doi:10.1038/s41439-021-00171-9)
Supplement: Supplementary file 3 — Supplementary Figure 3 [file 41439_2021_171_MOESM3_ESM.docx]

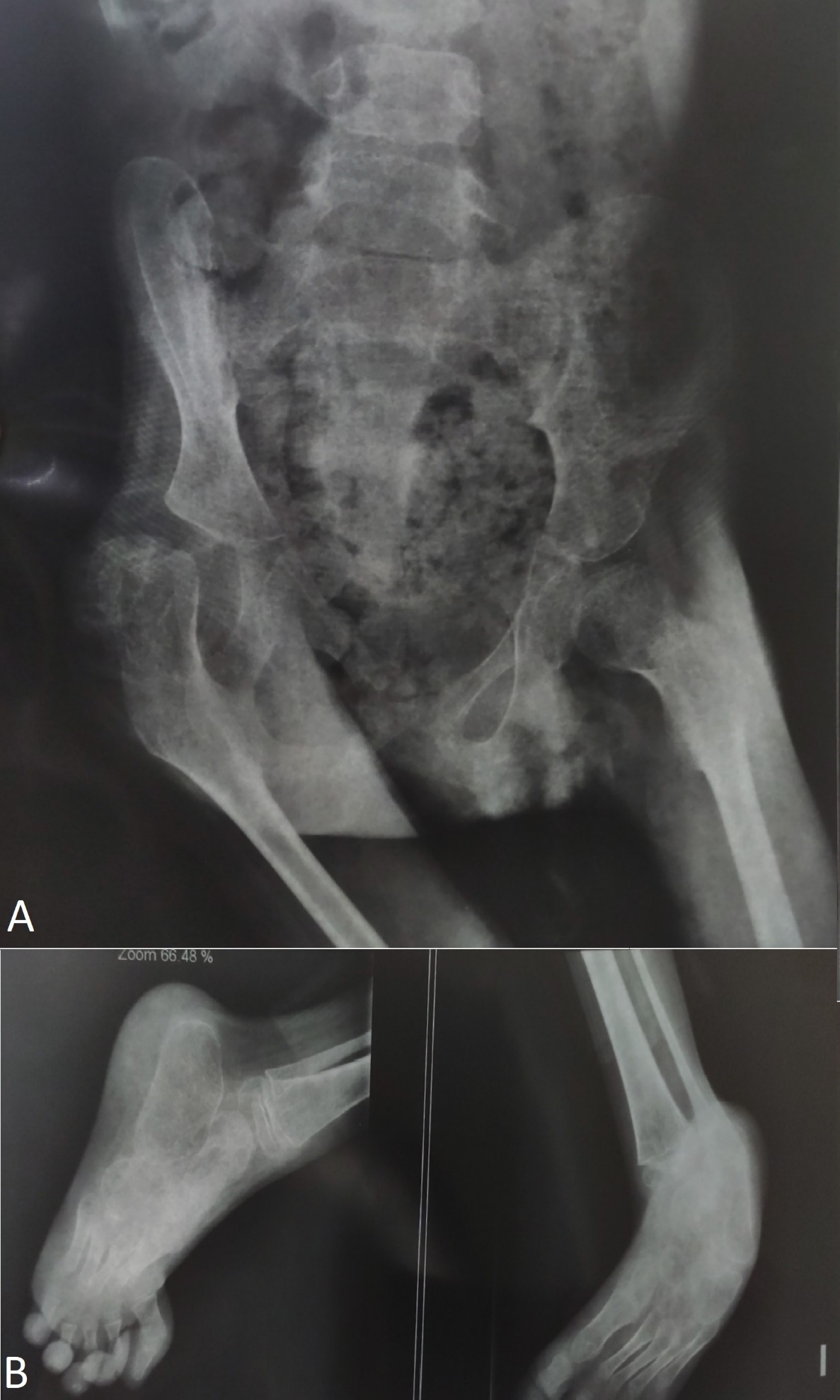


Supplementary Figure 3. X-ray radiography of the lower extremities and the pelvis of the proband of family 2. A; Right acetabulum agenesis, irregularities in the head of the left femur bone, and developmental dysplasia of the hip are present at the pelvis. B; Calcaneus, talus, navicular, cuboid bone malformations, inversion, malformation of tibiotalar joint, and first interphalangeal joint hyperflexion are present at foot radiography.
