# Supplementary figures and images for: Two novel Warburg micro syndrome 1 cases caused by pathogenic variants in RAB3GAP1
Source: Hum Genome Var. 2021 Oct 26;8:39. doi: 10.1038/s41439-021-00171-9 (PMC8548584; doi:10.1038/s41439-021-00171-9)

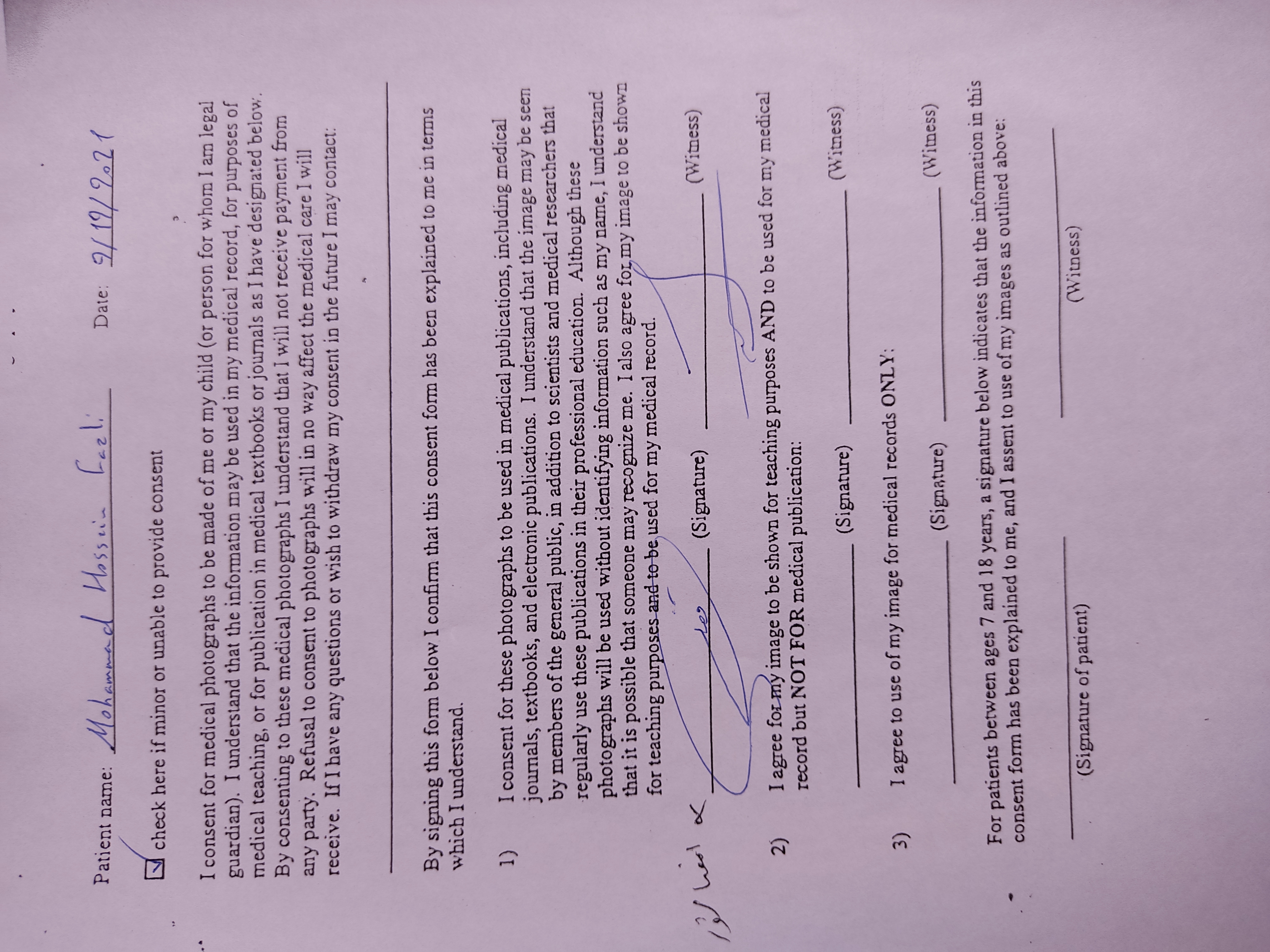

Supplement: Supplementary file 4 — Patient consent form 1 [file 41439_2021_171_MOESM4_ESM.jpg]

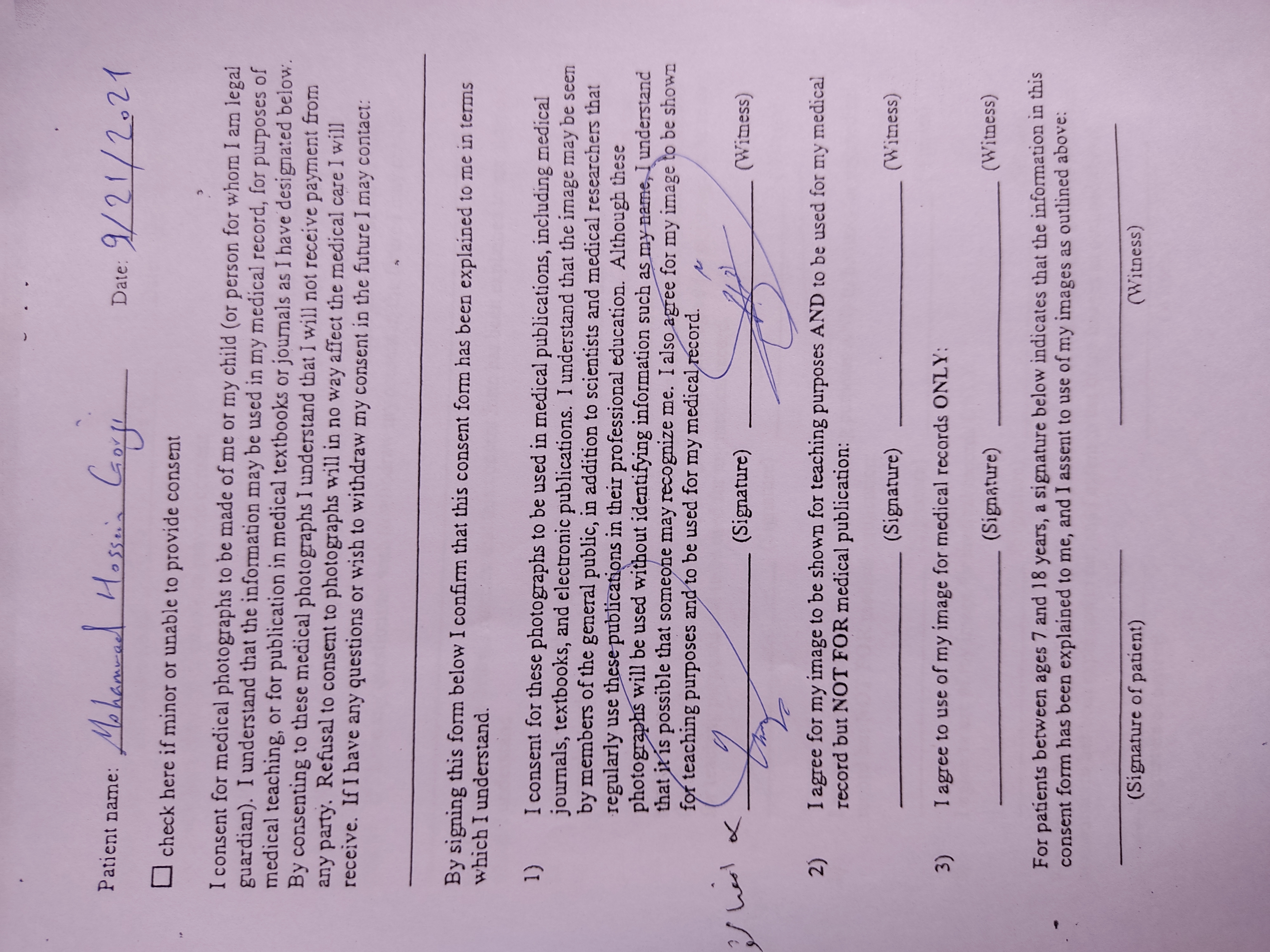

Supplement: Supplementary file 5 — Patient consent form 2 [file 41439_2021_171_MOESM5_ESM.jpg]
